# Assessment of nicotine for second hand smoke exposure in some public places in Romania by UPLC-MS

**DOI:** 10.1186/1752-153X-8-29

**Published:** 2014-05-03

**Authors:** Alina Bratan, Manuela Maria Mincea, Ioana-Rodica Lupşa, Marilen-Gabriel Pirtea, Vasile Ostafe

**Affiliations:** 1 West University of Timisoara, Timisoara, Faculty of Chemistry – Biology –Geography, Advanced Research Environmental Laboratories, Oituz str. 4, Timisoara 300086, Romania; 2 National Institute of Public Health, Regional Center of Public Health, Timisoara, Victor Babes str. 16, Timisoara 300226, Romania; 3 West University of Timisoara, Multidisciplinary Research Platform “Nicholas Georges - Roegen”, Oituz str. 4, Timisoara 300086, Romania

**Keywords:** Air, Nicotine, Cotinine, UPLC–MS, ETS

## Abstract

**Background:**

Air nicotine monitoring is a well-known procedure for estimation of exposure to second hand smoke. Few research studies were realized in Romania to evaluate environmental tobacco smoke (ETS) exposure of humans in different public places. The levels of airborne nicotine from environmental tobacco smoke and urinary cotinine and nicotine levels of some subjects were analyzed. In order to better implement/enforce the European legislation regarding the interdiction of smoking in the public places the national authorities need a rapid and reliable analytical method to quickly asses the state of the pollution with cigarette smoke of these populated areas.

**Results:**

The nicotine concentration in the air from different types of public buildings was determined. The median concentration of nicotine in the air from 32 pubs where the smoking was allowed was 590 ng · L^-1^, comparing with the pubs where the smoking was not permitted (22 locations) where the median concentration of nicotine was only 32 ng · L^-1^. Similarly, the median concentration of nicotine in restaurants where the smoking was allowed (23 locations) was 510 ng · L^-1^, in comparison with the restaurants where the smoking was prohibited (11 places) where the median value was 19 ng · L^-1^. The lowest concentrations of nicotine were found in high schools (8 locations, median concentration 7.4 ng · L^-1^), universities (5 locations, 23 ng · L^-1^) and hospitals (6 locations, 16 ng · L^-1^).

**Conclusions:**

The method was validated and gave good linearity, precision, accuracy and limit of detection. The buildings included hospitals, high schools, universities, pubs and restaurants. The presence of air nicotine was recorded in all buildings studied. The highest median levels of air nicotine were found in pubs and restaurants. The presence of air nicotine in indoor public buildings indicates weak implementation of the smoke free law in Romania.

## Background

Environmental tobacco smoke (ETS) is one of the most widespread carcinogenic exposures, being a class A carcinogen, and is considered a preventable occupational health risk. ETS is recognized to be an important risk factor for several chronic diseases such as lung cancer [1-3], coronary heart disease [4-7] and asthma [8-10]. Several epidemiological surveys have documented the link between second hand smoke (SHS) exposure and increased morbidity and mortality [11].

People have become aware of SHS exposure since the studies made during the 9th decade of the previous century [12]. The WHO Framework Convention on Tobacco Control promotes smoke-free environments to protect the health of nonsmokers from SHS [13]. The dissemination of the ETS monitoring studies results is very important especially for young people, because in the age group of 15 to 17 years, there are many persons who have a strong dependence on nicotine [14-16].

In Romania, since 2005 smoking in some public places is prohibited by the national law [17], that contains restriction of smoking in different areas such as public places, hospitals, high schools, workplaces, restaurants for nonsmokers, buses and in the vicinity of pregnant women and children [14]. The implementation of the law remained largely ineffective in the first seven years.

Measurement of nicotine is highly sensitive because it is a specific biomarker of tobacco smoke that represents the only possible source of nicotine in the air. Nicotine deposits almost entirely on indoor surfaces in a concentration of about 30 *μ*g · m^-3^, and persists for weeks to months [18,19]. Nicotine is rapidly metabolized, the half life time being about 2 h [20]. Cotinine, one of the major nicotine metabolite with a half life time of 20 h is frequently used for assessing tobacco smoke exposure and is typically detectable for several days (up to one week) after the use of tobacco. [20,21]. Cotinine can be measured in different body fluids or tissues including blood [22] urine, saliva, hair, [23] and teeth [24]. This bio-marker can differentiate the levels of exposure to tobacco smoke and levels of intake. The time needed to acquire increased levels of urinary cotinine is higher than 10 h after heavy ETS in a passive smoker [14].

The methods most used for the ETS monitoring are gas chromatography (GC) with flame ionization detection (FID) or nitrogen specific detectors [25], or GC coupled, with electron impact mass spectrometers (EI-MS) [14,23,24,26] as well as high performance liquid chromatography (HPLC) with MS detectors [27,28].

The existing surveillance system in Romania has no mechanism for routinely measuring exposure to SHS. There have been few studies or researches to look at exposure to SHS in different environments. In the current study, environmental measurements were used to characterize SHS exposure in key indoor public places in Romania. This paper presents an ultra performance liquid chromatography–mass spectrometric (UPLC–MS) quantitative method for nicotine levels in the indoor air, and for measuring the levels of airborne nicotine in some public houses, as a selective marker of tobacco smoke.

## Results

### Evaluation of the extraction solvent

Acetonitrile was selected as sampling solvent because it adsorbs/dissolves the highest quantity of nicotine, comparing with the other two solvents used (i.e. methanol and dichloromethane). Moreover, an additional dilution and/or evaporation step could be needed if using dichloromethane.

### Method validation

Aliquot samples containing 0.005, 0.02, 0.078, 0.313, 1.250, and 5 *μ*g · mL^-1^ nicotine in acetonitrile were used for the method validation.

The calibration graph resulted from the analysis of the calibration standard solutions prepared in acetonitrile was linear during the entire range of calibration solutions with a regression curve: y = 2.83 · 10^7^× - 5.51 · 10^5^ and a determination coefficient of 0.9998. The limit of detection (LOD) was 100 ng · mL^-1^ and the limit of quantification was 300 ng · mL^-1^ (for details of calculation see Additional file 1).

Precision was studied by collecting directly with the impinger the smoke from one cigarette and repeating the experiment 5 times. The average nicotine quantity found in the smoke collected during 2 minutes of suction at 0.8 L · min^-1^ was 1320 ± 60 *μ*g per cigarette (RSD 4.6%).

Accuracy was determined by spiking with reference standard solution (1.25 *μ*g · mL^-1^) 5 blank samples, i.e. collecting air from a well ventilated laboratory. These samples were treated as described in the *Sample Preparation* procedure. The data obtained were compared with theoretical concentration (i.e. 1.25 *μ*g · mL^-1^). Under these conditions the accuracy was expressed as percentage recovery: 89% (RSD 8.0%).

### Determination of nicotine in air and nicotine and cotinine in urine sample

In order to prove that there is a correlation between the quantities of nicotine inhaled as SHS at the working place, for two volunteers subjects (a non-smoker working in a pub where smoking was allowed and a non-smoker janitor from a hospital) the urine samples were also analyzed. Both of them have been working in their institutions for more than 6 months. The nicotine and cotinine levels present in their urine were considerably higher than the levels found in the urine of a technician (also non-smoker) working in a ventilated laboratory.

**Figure 1:**
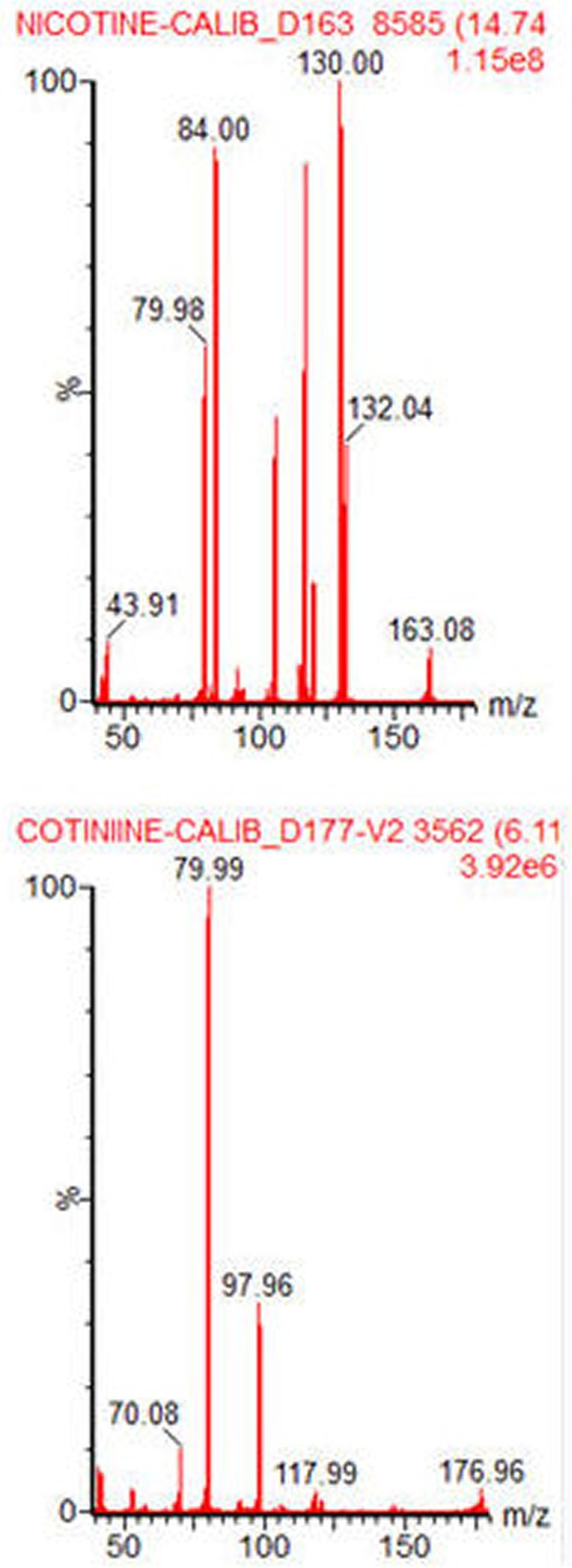
**Fragmentation mass spectra of nicotine (upper panel) and cotinine (lower panel) in the conditions mentioned in Table **1**.**

MS/MS optimized conditions for the Xevo TQD MS instrument are presented in Table 1. Figure 1 presents MS/MS spectra of nicotine (a panel) and cotinine (b panel). These spectra were used to select appropriate transitions for the quantification of these two analytes.

**Table 1:** Mass spectrometer parameters for nicotine and cotinine detection

**Analyte**	MRM transitions*	**Cone voltage (V)**	**Collision potential (V)**	**LOD (ng · mL**^**-1**^**)**	**LOQ (ng · mL**^**-1**^**)**
Nicotine	163 > 130 (117)	32	22	1	2.5
Cotinine	177 > 98 (80)	42	30	1	2.5

* m/z of precursor ion > m/z of the fragment used for quantification (m/z of the fragment used for confirmation).

As it can see from Table 2, there is a significant connection between the level of nicotine in the breathed air and the urine level of nicotine and cotinine, proving that the secondary smoke can be almost as dangerous as the primary/direct smoking.

**Table 2:** Correlation between SHS expressed as nicotine in inhaled air for 8 h and nicotine and cotinine level in urine

	**Barman in a Pub where smoking was allowed**	**Janitor in a hospital**	**Laboratory technician**
Nicotine in air (ng · L^-1^)	778	12	<LOQ
Cotinine in urine (ng · mL^-1^)	2241	34	<LOQ
Nicotine in urine (ng · mL^-1^)	373	5.7	<LOQ

**Figure 2:**
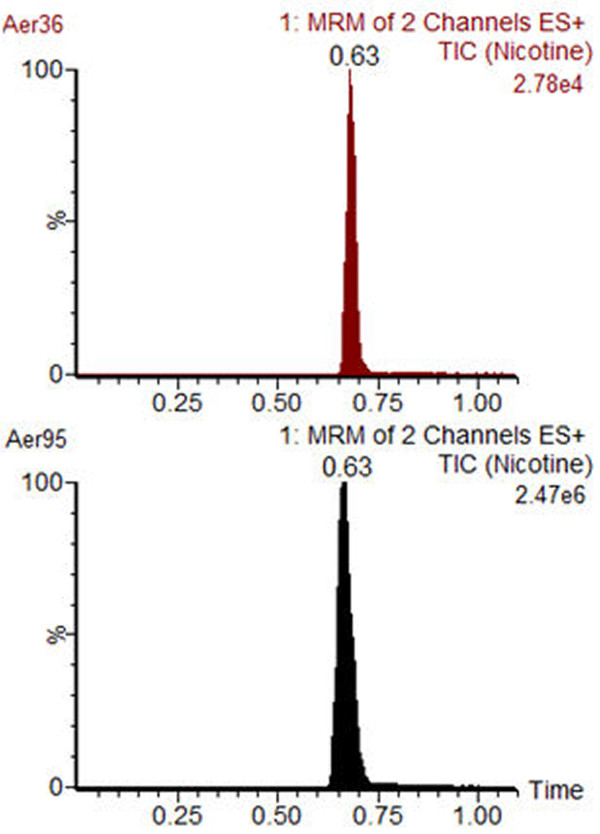
**Nicotine MRM (transition 163 > 130) chromatogram of the air sampled from a non-smoking pub (upper panel) and a smoking pub (lower panel).** The conditions are presented in Table 1.

As an example, Figure 2 shows two MRM chromatograms (transition 163 > 130) for nicotine analysis in air in a non-smoking pub (upper panel) and a smoking pub (lower panel). The difference between the nicotine content in those two environments, given by the scale in the top right corner, is of two orders of magnitude.

**Figure 3:**
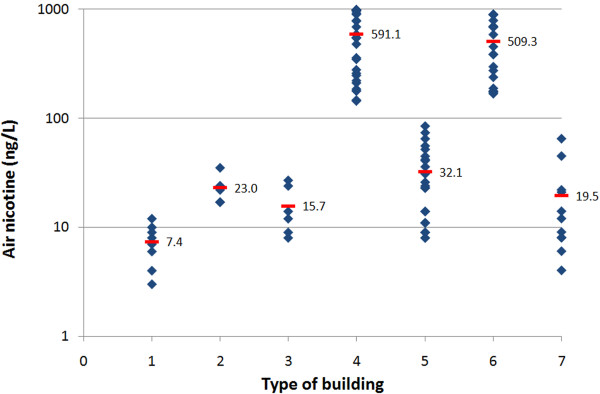
**Concentration of nicotine (ng · L**^**-1**^**) in different types of buildings: 1 = high schools (corridor), 2 = universities (corridor), 3 = hospitals (waiting rooms), 4 = Pubs (with smoking allowed), 5 = pubs (with smoking prohibited), 6 = Restaurants (with smoking allowed), 7 = Restaurants (with smoking prohibited).** The horizontal lines represent the median concentration.

Air nicotine concentrations (ng · L^-1^) in different types of buildings are presented in Figure 3. Buildings were monitored randomly at different hours during open program, in order to obtain unbiased samples. From the same building the sampling experiment was performed three times, in different days, at different hours and the results (presented in Table 3) were averaged. For this study high schools, universities, hospitals, pubs and restaurants were considered. For high schools and universities the samples were collected from the corridors situated at the main entrance. In the hospitals, the samples were collected from the main waiting rooms, in some cases this room being common with cafeteria. Regarding the eating-drinking places two categories of pubs and restaurants were studied: smoking and nonsmoking spaces.

**Table 3:** The levels of air nicotine in different building categories

**Building category**	**No. of buildings in the category**	Median concentration* (ng · L^**-1**^**)**	**Low (ng · L**^**-1**^**)**	**High (ng · L**^**-1**^**)**
High schools (corridor)	8	7.4	3	12
Universities (corridor)	5	23.0	17	35
Hospitals (waiting room)	6	15.7	8	27
Pubs (smoking)	32	591.1	144	988
Pubs (nonsmoking)	22	32.1	8	85
Restaurants (smoking)	23	509.3	169	897
Restaurants (nonsmoking)	11	19.5	4	65

*mean of three days.

As expected, the highest median levels of air nicotine were found in pubs, followed by restaurants, where the smoking was permitted (Table 3 and Figure 3). Measurable levels of air nicotine were found on corridors of high schools and of waiting rooms of hospitals.

## Discussion

UPLC-MS/MS is an appealing alternative to the GC assays due to the fact that sample preparation may be more straightforward than in GC–MS techniques [29] and because the UPLC separation method is more rapid than the GC method [14]. The UPLC–MS method presented for determination of nicotine concentrations in air is simple and rapid. The method validation gave the following values: 4.6% RSD precision and 8.0% RSD for accuracy. These results are comparable with the published LC methods for quantification of nicotine (and cotinine) although these analytical procedures were used for quantification of these compounds in serum [27] or urine [28].

This study shows that tobacco smoke is present in all public places examined from Timisoara. The study also demonstrated that in buildings where smoking is not allowed, levels of nicotine in the air are lower than in places where smoking is permitted but they are not zero. This implies that policies of having both smoking and non-smoking places are not entirely useful.

The nicotine concentrations found in pubs and restaurants where the smoking was permitted are very high. For someone who spent his time in such a place for 4–5 hours is equivalent to living with a smoker for one month. Similar results were mentioned by Nebot [30] and Hammond [31]. Comparing the results published by other studies, the concentrations of nicotine from Romanian pubs and restaurants where the smoking is allowed were higher than the majority of other public places [30,32-35], indication that either the law should not permit the smoking in any public place or these locations should have better ventilation systems.

## Conclusions

According with the published data of US Occupational Safety and Health Administration [36] a concentration of nicotine in the breathing air higher than 6.8 *μ*g · L^-1^ is considered to be “significant harm” for the humans exposed daily for more than 40 years. In this context, one may conclude that at least for employees and regular customers of Romanian pubs and restaurants where smoking is still allowed the risk to develop a lung cancer is one in 1000. In fact, all the analyzed pubs and restaurants where the smoking was allowed have higher concentration of nicotine in the air than the threshold level mentioned above. The World Health Organization stated that there is no proof for a not dangerous ETS exposure level [37] and our research confirmed that in many Romanian public buildings there are measurable levels of ETS exposure. The outcomes of this report indicate that properly implemented smoke-free strategies are necessary to reduce ETS exposure in indoor places.

Although the Romanian law prohibits smoking in all public buildings the enforcement of this law is very poor. This study may be considered as a scientific evidence for what the general public agree and feel, that new means should be envisaged by the authorities in order to really stop the smoking in the public places. Beside higher fees or other types of penalties applied to smokers and to the owners of the public places where the nicotine levels are found to be increased, educational programs regarding the health risks associated with tobacco smoke and benefits of smoking cessation should also be considered.

## Methods

### Materials

(S)-Nicotine ((S)-3-(1-Methyl-2-pyrrolidinyl)pyridine, Fluka N5511, 1 mg · mL^-1^ in methanol) and (S)-cotinine ((S)-1-Methyl-5-(3-pyridyl)-2-pyrrolidinone, Fluka C0430, 1 mg · mL^-1^ in methanol) were purchased from Sigma-Aldrich. All other solvents and reagents were of chromatography quality, purchased also from Sigma – Aldrich: ammonium formate (12466 Fluka), formic acid (06440 Fluka), dichloromethane (CHROMASOLV, 439223 Sigma), methanol (LC-MS CHROMASOLV, 34860 Sigma), acetonitrile (LC-MS CRHOMASOLV, Fluka 14261). HPLC grade water was prepared by SG Ultra Clear 2001-B Water Deionization System (Cole-Parmer) and additionally filtered through syringe filters PTFE 0,22 *μ*m (Teknokroma, Barcelona, Spain) immediately before use.

Working solutions 0.1 mg · mL^-1^ of each standard were made in 0.1% ammonium formate in 10% acetonitrile (buffered at pH 5.5) and from this, the calibration standards dilutions between 5 *μ*g · mL^-1^ and 0.005 *μ*g · mL^-1^ were freshly prepared before each analytical series.

### Sampling

For the current study, a survey was conducted across the city of Timisoara, sampling the air from different public places. The types of buildings considered for this study included hospitals, high schools, pubs, and restaurants. Sampling locations were selected to represent the areas where people frequently work or occupy. Second hand smoke was estimated by sampling of vapor-phase nicotine using an air pump (model HB3344-02 Tuff Personal Air Sampling, produced by Casella Cell, Badford, UK) coupled with an impinger (Pyrex glass Midget Impinger, from SKC Ltd, Dorset, UK) filled with 15 mL solvent (usually acetonitrile) for extraction/desolvation of nicotine from pumped air. The pump was set up to pump at a flow of 800 mL/min, for 15 minutes. Each building selected for this survey was monitored randomly at different hours during open program. The samples were collected in three different days and the results were presented as mean of the individual results.

For quality assurance and control purposes, a fixed sampling procedure was used to collect one blank sample for each of the 8–12 samples. For blanks, air samples were collected from a ventilated laboratory and they were handled and analyzed in the same way as the rest of the samples. The blanks were used to determine the method limit of detection and to calculate blank-corrected nicotine concentrations [38].

### Sample preparation

After passing 12 L air through pump (at a 0.8 L/min flow), the nicotine was extracted into acetonitrile (methanol and dichloromethane were also tested). The solvent was evaporated under gentle stream of nitrogen. The residue was re-dissolved in 100 *μ*L of mobile phase used for the equilibration of the analytic column. The samples were filtered on 0.2 *μ*m PTFE filters and afterwards 5 *μ*L of sample was injected into chromatographic system. By this procedure the initial samples were concentrated 150 times and in these condition the LOQ is 2.5 ng/L air collected.

The determination of nicotine and cotinine in urine samples, including collection, sample preparation, chromatographic separation and quantification are described elsewhere [39].

### Apparatus

UPLC–MS analyses were performed to determine the nicotine in air samples and urinary nicotine and cotinine. The chromatographical analysis was carried out on a Waters Acquity UPLC-MS system (Binary Solvent Manager, Xevo TQD equipped with an electrospray ionization interface) with a UPLC BEH Phenyl 1.7 *μ*m column (2.1 × 100 mm) using a gradient elution procedure. Mobile phase A consisted in 0.1% ammonium formate in acetonitrile and mobile phase B was 0.1% ammonium formate in 10% acetonitrile. The gradient profile was: 0 – 0.5 min, 100% B; 0.5 – 1 min, 95% B; 1 – 1.5 min, 0% B; 1.5 – 1.9, 0% B; 1.9 – 2 min, 100% B. The column temperature was set at 30°C. The analyses were run at a flow rate of 0.4 mL/min, and the sample volume injected was 5 μL. The ESI parameters for Xevo TQD MS detector were fixed as follows: capillary voltage at 3 kV, source temperature at 120°C, desolvation temperature at 450°C, and desolvation gas at 800 L/h. Nitrogen was used as the desolvation gas, and argon was employed as the collision gas. The detailed MS/MS detection parameters for each analyte are presented in Table 1 and were optimized by infusion of a 1 *μ*g mL^-1^ standard solution of each analyte into the detector at a flow rate of 10 *μ*L · min^-1^.

Analyses were carried out in multiple reaction monitoring mode, using two specific transitions for each analyte and the detector was fixed at maximum Extended Dynamic Range with peak mass widths of 2 and 1.5 amu for the first and third quadrupoles, respectively. The dwell time for all transitions was 0.01 seconds.

### Ethics

The study was approved by the Ethical Structures of the institutions to whom the authors are affiliated.

## Competing interests

The authors declare that they have no competing interests.

## Authors’ contributions

AB, MMM and VO have realized the sample collection, biological and chemical analyses and drafted the manuscript, MGP and IRL contributed to the conception and design of study and IRL contributed to data interpretation. All authors read and approved the final manuscript.

## Supplementary Material

Additional file 1 **Worksheet “Calib-curve” presents the data used to draw the calibration curve for nicotine.** Some extra-data regarding this experiment can be obtained from the worksheet “Dilutions”. The worksheet “Precision” presents the data used to calculate the precision of the analytical method. The worksheet “Accuracy” presents the data used to calculate the accuracy of the analytical method. Several worksheets were used as help the processing the data from the experiments of evaluation the nicotine concentration in the air collected from public places. The worksheet “AIR_SamplePrep” presents the summary of the sample preparation, “AirSamples” present the nicotine concentration, calculated from peak area, “AirSam-Calculus” and “AirSamp_Table” are intermediary calculations and “AirSamp_Fig3” is used to realize the Figure 3. The worksheet “UrineSamples” is used for the experiment of determination of nicotine and cotinine in the urine samples. The worksheet “Solvents” presents the data used to select the best solvent for sample preparation. Click here for file
